# Linking self-efficacy, entrepreneurial fit, family support, and entrepreneurial intentions: An explanatory mechanism

**DOI:** 10.3389/fpsyg.2022.959444

**Published:** 2022-12-21

**Authors:** Ishfaq Ahmed

**Affiliations:** Hailey College of Commerce, University of the Punjab, Lahore, Pakistan

**Keywords:** entrepreneurial self-efficacy, family support, entrepreneurial intention, students, person-environment fit

## Abstract

Entrepreneurial ventures are outcomes of favorable internal and external factors. But the outcomes are always uncertain, often creating a situation of cognitive/perceptual dismay. One of such perceptual aspect of entrepreneurship that has recently emerged is person-entrepreneurship fit (P-E fit). By using this underlying aspect, this study entails investigation of its antecedents (entrepreneurial self-efficacy) and consequences (entrepreneurial intentions) in presence of boundary condition of family support. Data is collected through a structured questionnaire from 359 students enrolled in the last semester of their graduate and postgraduate programs at three large public sector universities. The findings of the study reveal that entrepreneurial self-efficacy influences perceptions of P-E fit and entrepreneurial intentions, while P-E fit works as a partial mediator. This study also found that family support is an important boundary condition that influences the relationship of self-efficacy and P-Ent fit.

## Introduction

Entrepreneurship is a drawn-out process (Moroz and Hindle, 2012) and uncertainty is a permanent part of it (Knight, 2002; Hsu et al., 2019). It is therefore believed that entrepreneurial activities are the outcome of both internal dispositional and individual manifestations toward entrepreneurship (i.e., intentions), the ultimate determinant of entrepreneurial behaviors (Ahmed and Islam, 2021). While looking at the various determinants of entrepreneurial intentions, it is expected that not everyone seems to be suitable for entrepreneurial career and a specific form of fit between individual – entrepreneurial career is required (Gupta et al., 2009; Hsu et al., 2016). So the value of perceived fit increases, even before starting a business ventures; as such perception may influence entrepreneurial actions and behavior. Fit has a direct bearing on the nature of entrepreneurial outcomes (Markman and Baron, 2003), and its value is even cherished in the relationship of personal dispositional factors and intentions. Hsu et al. (2019) seminal work on P-Ent fit also highlighted that how fit influences the entrepreneurial intentions and motives before individual engagement needs more attention as P-Ent fit may influence one’s level of involvement in such cumbersome entrepreneurial activities, but how fit perceptions are made or what factors influence one’s fit perceptions is an area that has not gained due attention.

Against this backdrop, this study aims to investigate the determinants of P-Ent fit. A profound look at literature highlights that P-Ent fit framework extends the person-environment fit (P-E fit) theory to entrepreneurship (Markman and Baron, 2003), and assumes that individual attitudinal and intentional outcomes are shaped by P-Ent fit perceptions. But it is observed that the how fit perceptions may arise and how they may lead to entrepreneurial intentions and actions is largely underinvestigated topic in the field of entrepreneurship (Hsu et al., 2019). While identifying various contributory factors of such manifestations, personality is found to be the most important, whereas entrepreneurial self-efficacy (ESE) is the most widely agreed and valued trait (Ahmed et al., 2021; Neneh, 2022). ESE is a personality trait that is considered as a promising dimension of entrepreneurial self (e.g., Ciuchta and Finch, 2019; Hsu et al., 2019; Ahmed and Islam, 2021). But the results explaining the outcomes of self-efficacy are either not clear or offer mixed results (Laguía et al., 2019; Anwar et al., 2022), thus highlighting the need of some peripheral variables that may influence possible consequences of ESE.

We contemplate that the external variable (family support) may play a pivotal role in explaining the outcomes of ESE. The same could be assumed on the fact that perceived P-Ent fit is based on one’s belief about his/her suitability for entrepreneurial ventures, the belief may be influenced by both internal and external factors. As the P-E fit theory believe that human behavior is influenced by both personal and environmental factors and their collective influence is always synergistic (van Vianen, 2018); we assumed that individual variables (i.e., ESE) and external variables (i.e., family support) both can synergistically influence the fit perceptions of entrepreneurs.

**Figure 1:**
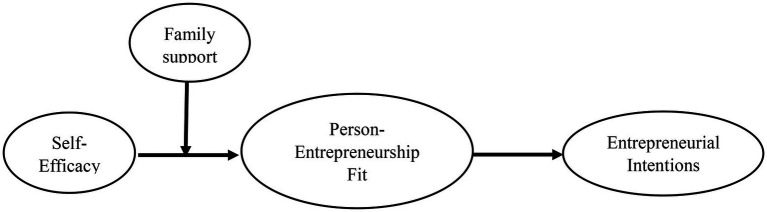
Conceptual model.

Similarly, while looking at the determinants of one’s fit perceptions, van Vianen (2018) considered person-job and person-organization fit as P-E fit slices and concluded that environment and individual factors both are important for their prediction. Player et al. (2017) witnessed that fit is influenced by external forces (e.g., leadership) and offers individual-level outcomes in exchange. Thus assuming the role of both individual and external environmental factors (Wang and Wang, 2018), in tandem, in predicting fit and its outcomes is considered viable and theoretically. Furthermore, studies on self-efficacy and family support and their predictive ability of one’s p-ent fit are also scant. Thus, the study offers a novel explanation in predicting fit perceptions of entrepreneurs, through personal dispositions (i.e., ESE) and conditional factors (FS; see Figure 1, the conceptual model).

Considering the strengths of family support it is believed to be a variable of utmost importance. It is the family that may develop confidence and self-belief of an entrepreneur, by nurturing attitude, emotions, behavior, and personality of its members and often set directions for offspring (Stamboulis and Barlas, 2014). Defiantly, the absence of such support may hamper one’s views of being fit for entrepreneurship. As Pakistan is country with joint family system, and wide span of payors than earners, the career choice is also influenced by the family (Iftikhar, 2016; Ahmed and Islam, 2021), and entrepreneurship is only believed to be an option when there is no job available (necessity entrepreneurship, GEM, 2019). Thus it would be an interesting aspect to study, in the Pakistani context, as the prevailing issue is largely been ignored and even literature is inaudible on every such issue. Based on the explained premise, we assumed that the absence of family support may even hamper the fit perceptions of individuals who have high-self efficacy but not support from family to start business. This family support may intensify or condense the link of self-efficacy and perceived p-ent fit.

This study, thus, is distinctive in two ways; first, it covers the role of ESE in predicting one’s fit perception and further its influence on entrepreneurial intentions. Secondly, it covers the moderating role of family support, as it may influence the link between ESE and P-Ent fit ultimately making us assume the mechanism between the variables of interest. Thirdly, this current study adds in person-entrepreneurship fit (P-Ent fit, Markman and Baron, 2003) literature by assuming the role of both ESE and family support. Fourthly, the findings of the study are based on the sample taken from a highly collectivist society where career choices and decisions are often influenced by the family and other social actors (Ahmed et al., 2021).

## Theorization and hypotheses development

### Underpinning theory

This study is built on the premise of Person-Environment (P-E) fit which proposes that people consider and try to find a fit between their personal characterisitcs and their environment (van Vianen, 2018; Liu et al., 2022). The fit may be assumed at various levels including fit with individuals, groups, organization, or occupation. Individual characterisitics may include their biologoical, physical, physiological and psychological traits, while environment may cover job roles, cultural traits, characteristics of other individuals and social environment. In order to find a fit or misfit individuals evaluate their environment and find the congruence between them and environment, which may arise in two forms, i.e., supplementary/need supplies and complementary/demand abilites. The former includes a fit perceptions when one believes that environment possess the ability to meet the needs of individuals, while the later deals with one’s perceptions that environment lacks some traits and those are off-set by the individual him/herself or vice versa (Muchinsky and Monahan, 1987). Thus both these congruence aspects either ensure a fit or find a way to create it (van Vianen, 2018). One’s fit with the supervisor, team and organization is a form of supplementary fit, while the job or occupation fit are forms of complementary fit (van Vianen, 2018; Gander et al., 2020). When the environment can meet one’s psychological needs, the individual tends to feel fit with that (Liu et al., 2022). Based on the given lines, this study proposes that one’s feelings of fit with environment may influence their career/occupation fit perceptions. This study proposes that personal factors (i.e., ESE) enhances one’s perception of fit with the career of entrepreneurship (supplementary fit), while the support from the family fills the gaps or covers the deficiency or vice versa (complementary fit). It further suggests that one’s attitude and behavior is influenced by both internal and external factors (Saks and Ashforth, 1997; Kristof‐Brown et al., 2005). While taking this presumption to the P-Entrepreneurship fit framework (Markman and Baron, 2003) we assume that family support, being the external factor, will influence the relationship between ESE and P-E fit as individuals with high family support will feel confident and have high perceptions of self-fit with entrepreneurship. The following section covers the hypotheses developed based on the given theoretical premise and literature.

### Hypotheses development

Entrepreneurial process is influenced by various dispositional factors and personality is one of those (Ciuchta and Finch, 2019; Laguía et al., 2019). A profound look at literature signifies that entrepreneurial self-efficacy (one’s belief in his/her abilities; for entrepreneurs its henceforth ESE), is one of the most promising traits (Crespo et al., 2018; Hussain, 2018). Individuals’ with stronger ESE are found to display a higher level of venturing intentions (Chang et al., 2019; Pérez-López et al., 2019). A recent meta-analysis on entrepreneurial intentions and behavior also found that ESE is one of the most significant predictors (Newman et al., 2019), because it influences employees at their cognitive and emotional levels (Fuller et al., 2018; Abdi and Pak, 2019). The same has been probed by another meta-analysis of Barańczuk (2019), who observed that personality dimensions have a direct bearing on the emotional regulation strategies. Schindler and Querengässer (2019) further found that various dimensions of personality (e.g., neuroticism, openness to experience) has a direct bearing on the ways of handling emotions (Schindler and Querengässer, 2019). Pollock et al. (2016), while giving a detailed description of personality and emotions link, found that positive personality aspects influence the emotions more sturdily than the negative aspects. Like all other personality traits, it is believed that ESE (a positive dispositional factor) may also increase one’s potentials of channelizing efforts toward business ventures (Markman et al., 2002). But how one’s fit perceptions work as mechanism between this relationship is an area that has not gained due attention.

One of the under-investigated mechanism is through P-Ent fitness. The fit perceptions are largely influenced by the belief about the extent to which one’s needs are met by the entrepreneurial venture. The Markman and Baron’s (2003) seminal work on P-Ent highlights that venturing intentions and behaviors are important outcomes of fit perceptions. There are two dimensions of P-Ent fit, i.e., need supplies and demand abilities (Hsu et al., 2019), where the former focuses on fulfillment of the needs that determine one’s attitude and behavior (Higgins, 1998; Avey et al., 2009; Greguras and Diefendorff, 2009). For instance, one’s belonginess needs would make him/her find a workplace that could be termed as home (Avey et al., 2009), thus the actions would be influenced by such needs. On the other hand, the later focuses on one’s belief on his/her abilities to perform desired task effectively (Greguras and Diefendorff, 2009). According to Hsu et al. (2019) the same can be replicated to entrepreneurial settings where one’s belief in his/her abilites (a concept parallel to entrepreneurial self-efficacy, ESE) can determine entrepreneurial attitude and behavior.

The presumption could be supported by the fact that entrepreurs work to fulfill their needs of achievement through venturing actitivites, while job seekers focus on fulfilling their (financial, affiliation and self-achievement) needs through job (Cable and Judge, 1996; Saks and Ashforth, 2002; Westerman and Cyr, 2004). Individuals with high security needs tend to remain away from entrepreneurial actions due to uncertainity attached to that (Knight, 2002; Shane, 2008), while those who strive for high-achievement needs tend to take risk of starting business (Baron, 2004). Therefore, it is believed that one’s needs and beliefs (ESE) may influence their fitness perceptions about entrepreneurial activities. According to Bandura and Walters (1977) self-efficacy is a belief system that determines one’s course of actions. Past studies have found that individuls with high ESE tend to indulge in business activities (Chang et al., 2019; Newman et al., 2019; Pérez-López et al., 2019), because they feel that they are fit for such acts. As ESE and demand-abilities perspectives are closely associated (Greguras and Diefendorff, 2009; Hsu et al., 2019), it is to assume that P-Ent fitness could be an outcomes of such a belief or demand-ability. The same is hypothesized below:

*H1:* Individuals’ with high entrepreneurial self-efficacy will perceive more P-Ent fit and will have higher entrepreneurial intentions.

Past studies have shown that it’s not merely the dispositional factors that influence entrepreneurial intentions, but certain external factors that determine entrepreneurial intention. The value of such factors has been highlighted in past literature. For instance, the past studies highlighted the value of studying conditional variables between intentions and its predictors (Schlaegel and Koenig, 2014; Ahmed et al., 2021). One of such conditional variables is family support (Neneh, 2019). Family forms an important part of the determination of one’s attitude and behavior, as it can influence one’s confidence, idea creation ability, and belief of starting a venture. Parents and families influence the career choices of individual members through emotional and attitudinal responses. Parents are also carriers of family values thus may set directions for its members (Rachmawan et al., 2015). According to Stamboulis and Barlas (2014) family influences entrepreneurship by determining their emotions, attitudes, behaviors, and personality and therefore set directions for offspring (Stamboulis and Barlas, 2014). Empirical literature also highlights that family size, structure and formation influences decision-making skills, and career choices of its members (Lotfizadeh and Heidarzadeh Hanzaee, 2014). Edelman et al. (2016) highlighted that family support is most important at idea generation and start-up phases than the growth and development phases, as new ventures need moral and emotional support. As the family support encourages one to convert intentions into behavior (Tolentino et al., 2014; Edelman et al., 2016), its absence may have detrimental effects on such intentions.

Support from family is based on its behavior which is largely influenced by the economic conditions as high-income countries have been witnessed to have more start-up ventures than low-income countries (Henrekson and Sanandaji, 2014). Nevertheless, the social and economic structures (joint family system, number of breadwinners and number of family members to feed) put more pressure on its members (e.g., fresh graduates) to start earning by finding a job rather than putting effort on the timely and costly process of entrepreneurship (Ahmed et al., 2021). Culture is also found to influence career choices, as it influences at both macro and micro levels (Hoyte, 2019). At micro-level it determines family roles toward business ventures (Annink et al., 2015), while at macro level it determines the family culture which can influence entrepreneurial actions (Lee and Peterson, 2000). As low-income countries lack infrastructure and resources to facilitate entrepreneurs (Henrekson and Sanandaji, 2014), it is expected that entrepreneurial ventures will not get support at a micro (family) level in developing countries. Panda (2018) also inferred that lack of business, economic and political (BEP) culture creates hindrances for entrepreneurial ventures. Pakistani environment is found to be largely unsupportive for entrepreneurship, as Iftikhar (2016) commented that the family and social structure of the Pakistani environment hinders entrepreneurship. Moreover, graduates are influenced to find a job instead of focusing on entrepreneurial ventures. GEM’s (2012) report also supports the fact as entrepreneurship in Pakistan is necessity driven where it’s merely aimed to fill the needs created due to the non-availability of job.

Considering the family role in P-Ent fit literature, this study assumes that family support can influence the relationship between ESE and P-E fit. This could be inferred because P-E fit theory assumes that one’s attitude and behavior is influenced by both internal and external factors (Saks and Ashforth, 1997; Kristof‐Brown et al., 2005). From P-Ent fit framework (Markman and Baron, 2003) we assume that family support, being the external factor, will influence the relationship between ESE and P-E fit as individuals with high family support will feel that their psychological needs are met by the family which will result in increased entrepreneurial fitness perceptions. This relation could be assumed on the grounds as the family may provide confidence, courage and emotional support to the individual to start a business (e.g., Lotfizadeh and Heidarzadeh Hanzaee, 2014; Stamboulis and Barlas, 2014; Tolentino et al., 2014; Edelman et al., 2016), thus it could also be presumed that in presence of family support individuals will feel greater fit with entrepreneurial activities because the family will provide more support and encouragement to take the risk. Based on the discussion generated above following hypotheses is created:

*H2:* Family support will strengthen the relationship between entrepreneurial self-efficacy and person-entrepreneurship fit such that the relationship would be stronger when support level would be high and vice versa.

## Research methods

### Participants and procedure

Data for this study was collected form 359 students enrolled in the last semester of graduate and postgraduate level programs in large public sector universities of Pakistan. Such students are likely to make career choices in near future as have had exposure to almost 40 business subjects and they would be open to practically using their learning (Ahmed et al., 2021). University students are a good sample for entrepreneurial research as their intentions are most important as they have to make career choices shortly (Hsu et al., 2017). The selection of undergraduate students is also justified as such students are often found to have no business experience that may influence their intentions (Arentz et al., 2013), and education and skills are important determinants of their career choices and success (Frunzaru and Cismaru, 2021). The sample drawn from developing country could also provide insightful findings as the literature highlights that the career choices are made by families rather than individuals (Iftikhar, 2016; Ahmed et al., 2021).

Students were approached at two points of time with 6 weeks interval. This approach is considered as a source of overcoming issues of common method biasness (Podsakoff et al., 2003). Self-sampling approach was used to access the students who were willing to be participant of the study (Shin et al., 2022). At first point of time 487 students participated and filled their responses for entrepreneurial self-efficacy and family support. At second instance, only 376 students were available to respond against their E-fit perceptions and entrepreneurial intentions. 17 of the responses were carelessly filled (incomplete) and thus considered reduendent. Most of the respondents were male (73.25%), business graduates (68.45%), with no family background business (93.25%), undergraduate students (76%) and single earning member (87.25%).

### Measures

Data was collected through a structured questionnaire adapted from previous studies. Entrepreneurial self-efficacy was operationalized with Cox et al. (2002) 10 items scale with sample item “How much confidence you have in your ability to plan a new business?” The scale has been widely used in the past and found reliable (Kickul et al., 2007; Kazumi and Kawai, 2017). The scale of family support was taken from the work of Shen et al. (2017) containing five items. Person-entrepreneurship fit was taken from the scale of person-job fit (P-J fit). P-J fit is people believe that they have those abilities that meet the requirements of the job, thus ensuring the individual compatibility with a job (Ballout, 2007). It is also highlighted in the literature that an individual should also have an organization (P-O fit) and culture fit (P-C fit; Ballout, 2007), but we assumed that these two dimensions may not have as generalized applicability as P-J fit could have. This could be attributed to the conceptual definitions of the concepts, as P-O fit deals with one’s compatibility with organizational values and P-C fit deals with one’s compliance with the culture of an organization (Parkes et al., 2001; Van Vienen et al., 2004). As entrepreneurial ventures are the outcome of entrepreneurs’ dispositional efforts and both the value system and culture is articulated by entrepreneur the existence of fit would be natural. Moreover, both these forms of fit will exist after the business venture starts its operations, thus while considering intentions, the P-J fit could be considered the most suitable aspect of fit. This study used Tseng and Yu’s (2016) P-J fit scale containing three items. Entrepreneurial intentions were measured through Liñán and Chen (2009) three items scale. All the aforementioned measures were widely accepted in literature and used in various studies.

## Findings

Data analysis was carried out in two phases, where the first phase covered preliminary analysis followed by the use of structural equation modeling and multiple regression to test hypotheses in the second phase. The guidelines given by Kline (2005), Tabachnick et al. (2007), Hair et al. (2010), and Byrne (2012) were used for preliminary analysis (which covers test for missing values, multicollinearity, normality, outliers). These tests are mandatory to have factual results in the following stage. As the data was collected through the personally administrated questionnaire the chances of missing values were not present and were ensured by the analysis. Correlation coefficient results highlighted that independent variables were not strongly correlated (*r* < 0.85), thus the issue of multicollinearity was not severe. Data was also found to be normal as the values for skewness and kurtosis were within acceptable limits (<±1 for skewness and <±3 kurtosis).

Though the researcher used methodological approach (two lags approach), to overcome issues of CMV, the same was assessed using Harman’s single factor score. The single factor explained 28.5% variance which was below the threshold of 50% (Podsakoff et al., 2003), thus the CMV was not severe. Furthermore, the four factors model had the suitable fitness indices (*χ*^2^/df = 1.59, CFI = 0.90, RMSEA = 0.04, SRMR = 0.08) highlighting the absence of issue of CMV.

After preliminary analysis descriptive statistics and correlation analysis was done. Findings of both the analysis are provided in Table 1. It is evident from the table that ESE is positively associated with person-entrepreurship fit (*r* = 0.39, *p* < 0.05), entrepreneurial intentions (*r* = 0.32, *p* < 0.05) and family support (*r* = 0.09, *p* < 0.01). Furthermore, person entrepreneurship fit is also significantly associated with entrepreneurial intentions (*r* = 0.43, *p* < 0.05) and family support (*r* = 0.23, *p* < 0.01), while family support was also associated with entrepreneurial intentions (*r* = 0.27, *p* < 0.01). It is thus to assume that all the variables of the study are related, which help us move further with data analysis.

**Table 1:** Correlation and reliability analysis.

Variables	1	2	3	4	Mean	SD	*α*
1-ESE	1				4.43	0.45	0.89
2-P-E fit	0.39*	1			4.05	0.87	0.83
3-EI	0.32*	0.43*	1		3.98	0.87	0.82
4-FS	0.09**	0.23**	0.27*	1	2.99	1.79	0.91

ESE, entrepreneurial self-efficacy; P-E fit, person-entrepreneurship fit; EI, entrepreneurial intentions; FS, family support; *α*, Cronbach Alpha; **p < 0.01, *p < 0.05.

We conducted two stages of structural equation model analysis with the help of AMOS. In the first-stage CFA (i.e., Confirmatory Factor Analysis) was done which was aimed to find the loading of each factor on the latent variable (Hair et al., 2010). Model fitness criteria of Williams et al. (2009) were used and it was found that the CFA model was fit (i.e., *χ*^2^/df *= 2.45,* RMSEA = 0.043, CFI = 0.95, SRMR = 0.067). It was also found that the factor loading of each factor was above the threshold value of 0.5 (ranging from 0.52 to 0.79; Hair et al., 2010). Additionally, the values of average variance extracted and composite reliability were also above the threshold value (i.e., 0.50 and 0.60 respectively), thus the measures have convergent and discriminant validity. In the next stage of data analysis measurement model was also found to be fit (i.e., *χ*^2^/df *= 2.33,* RMSEA = 0.053, CFI = 0.94, SRMR = 0.066). Discrimiant validity was further assessed using Fornell and Larcker (1981) approach, the results of which are shown in Table 2, which shows that the measures are discriminatnly valid.

**Table 2:** CFA and validity.

		Loadings	CR	AVE	MSV	1	2	3	4	5
1	ESE	0.52–0.76	0.798	0.609	0.428	0.758				
2	P-Ent fit	0.56–0.68	0.810	0.674	0.425	0.542	0.733			
3	Ent. Intentions	0.59–0.70	0.795	0.597	0.441	0.385	0.508	0.746		
4	Family support	0.56–0.79	0.892	0.655	0.475	0.528	0.452	0.248	0.726	

Hypotheses testing is done through Hayes Process Macros and SPSS. Results of hypotheses testing are provided in Table 3. The analysis covers bootstrapping and Sobel tests, and its results are shown in Table 3. It is evident that entrepreneurial self-efficacy significantly influences P-E fit (*β* = 0.34, *p* < 0.05) and entrepreneurial intentions (*β* = 0.39, *p* < 0.05). The indirect effect of self-efficacy on entrepreneurial intentions tested through a Sobel test also highlights that the results are significant (Sobel z = 5.01, *p* < 0.05). Bootstrapping results for mediation also highlight the fact that indirect effects are significant as it does not contain zero (0.05, 0.16 at 5,000 bootstrap sample), thus H1 was supported and partial mediation was proved.

**Table 3:** Regression results.

	*β*	SE	*t*	Value of *p*		
ESE – EI	0.39	0.13	4.04	0.00		
ESE – P-E fit	0.34	0.10	3.98	0.00		
P-E fit – EI (controlling of ESE)	0.29	0.15	5.06	0.01		
ESE – EI (controlling AR)	0.20	0.09	3.99	0.00		
Indirect effects and significance using normal distribution sobel	**Value**	**SE**	**L95%CI**	**U95%CI**	***z***	***p***
	0.19	0.07	0.04	0.20	5.01	0.01
Bootstrap results for indirect effects	***M***	**SE**	**L95%CI**	**U95%CI**		
	0.14	0.06	0.05	0.16		

Bootstrap sample size 5,000; L, lower limit; U, upper limit; CI, confidence interval.

**Figure 2:**
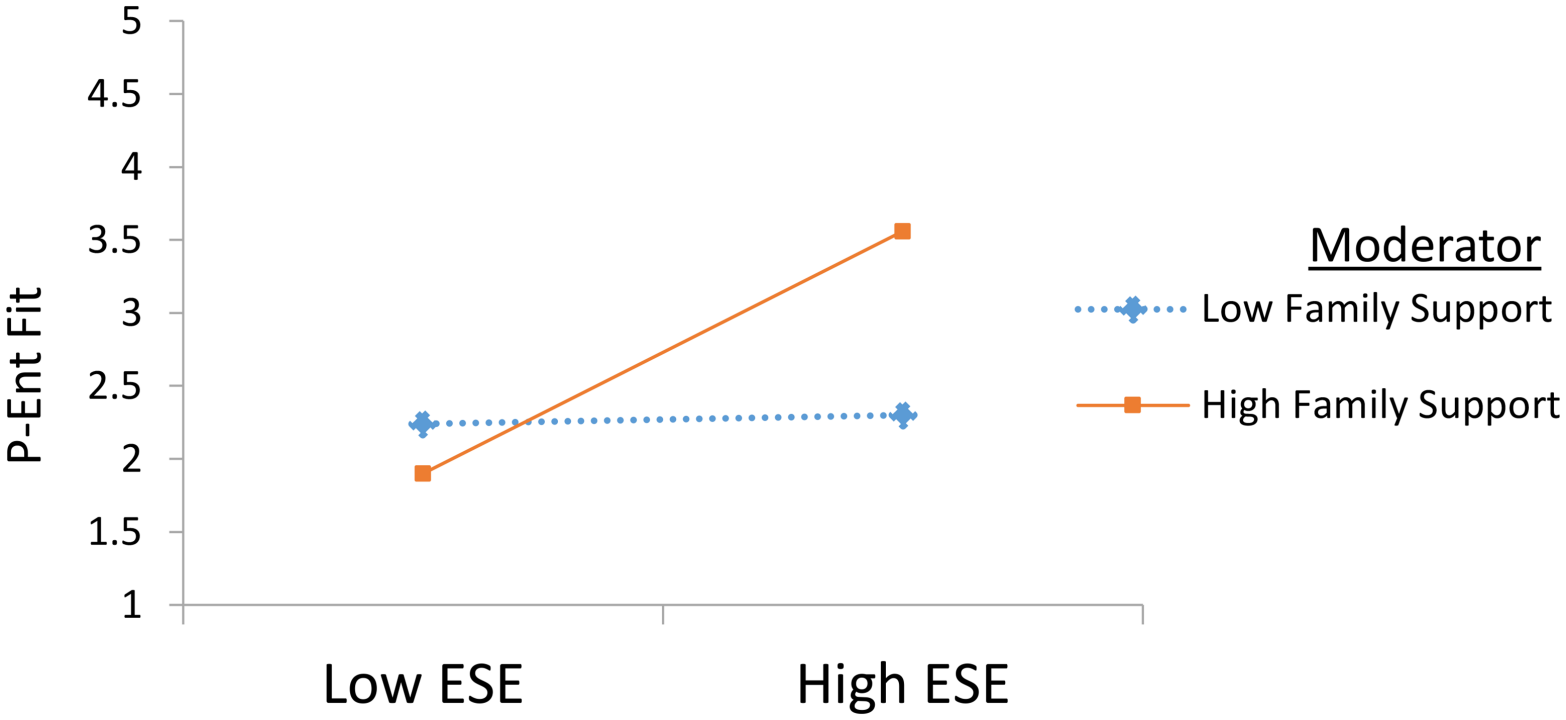
Slope of moderated mediation.

Moderation analysis (H2) results are shown in Table 4 and Figure 2. Table 4 reveals that entrepreneurial self-efficacy and family support interaction significantly influences P-E fit (*β* = 0.51, *p* < 0.01), and this effect is greater than the direct effect of entrepreneurial self-efficacy on P-E fit (*β* = 0.34, *p* < 0.01), thus highlighting the fact that family support strengthens the relationship between entrepreneurial self-efficacy and P-E fit (H2 is supported). Furthermore, all the demographical variables (age, gender, entrepreneurial experience, and family business background) either do not or have a weak influence on P-E fit, thus there was no need to control them.

**Table 4:** Moderation results.

**Variables**	**P-E Fit**
Age	0.04
Gender	0.06*
Entrepreneurial experience	0.09
Family business	0.04*
Entrepreneurial Self-Efficacy	0.34*
Family support	0.19*
Entrepreneurial self-efficacy × family Support	0.51*
Adjusted *R*^2^	0.23**

**p < 0.05; *p < 0.01.

## Discussion and conclusion

This research endeavor extends the literature on entrepreneurial self-efficacy (ESE) and intentions by bridging them through person-entrepreneurship fit (P-E fit) as mediator and family support as a moderator. The hypothesized model was evaluated through two hypotheses, where the results supported both of them. The findings of the study proved that ESE significantly predicts entrepreneurial intentions and P-E fit perceptions. Moreover, it was also found that P-E fit works as a partial mediator in the association of ESE and intentions (supporting H1). Literature is also evident of the fact that ESE significantly predicts intentions (e.g., Crespo et al., 2018; Fuller et al., 2018; Hussain, 2018; Shahab et al., 2018; Chang et al., 2019; Newman et al., 2019; Pérez-López et al., 2019), while P-E fit relation with ESE was either ignored or not given due attention. Based on the demand-abilities/complementary dimension of person-environment fit (Hsu et al., 2019), this study found that one’s belief in his/her abilities positively and significantly influences perceptions of fit. The results could be elaborated because past studies (e.g., Chang et al., 2019; Newman et al., 2019; Pérez-López et al., 2019) have shown that individuals with high ESE may have more trust in themselves and believe that the job is fit for them (i.e., Perception of P-E fit). Our study thus provides a shred of empirical evidence linking self-efficacy and P-E fit, which has been valued in the past but lack empirical support. These findings thus add value in the literature on the person-environment fit theory for entrepreneurship (Markman and Baron, 2003), which assumes that the P-E fit offers positive outcomes. While our study offers evidence covering both antecedents and consequences of such fit.

This study also considers family support roles in the mechanism of ESE and P-E fit. Family support has been observed to have a direct impact on career choices, earning patters and vocational behaviors (Ogawa and Ermisch, 1996; Iftikhar, 2016; Ahmed et al., 2021). Poor and developing countries with low *per capita* income are found to have an unsupportive environment for entrepreneurship (Henrekson and Sanandaji, 2014), due to large family size and less earning hands. As the sample of the study was drawn from the students enrolled in large public sector universities of Pakistan, it is assumed that the effects of low-income families would be prevalent. Iftikhar (2016) commented that in Pakistani family setup students are often forced to start the job immediately after completion of their degrees, thus entrepreneurial intentions remain either low or not converted to the actual behavior. Furthermore, the labor force participation rate in Pakistan is found to be influenced by demographical variables and cultural traits (Hussain et al., 2016). These cultural and family traits make entrepreneurship a secondary choice instead of primary career choice (Lee and Peterson, 2000; Annink et al., 2015; Hoyte, 2019); as GEM indicators also highlighted that entrepreneurship is a necessity driven practice, which is only an alternate of job, in Pakistan (GEM, 2012). It was thus to assume that family support would be an important predictor of one’s entrepreneurial intentions and fit perceptions.

The results of the study proved that family support influences the relationship between ESE and P-E fit (supported H2). Our findings thus prove that when individuals receive more support from the family the perceptions of fit due to ESE are increased, thus family support buffers the said relationship. The results are consistent with past studies; for instance, Crespo et al. (2018) found that ESE itself is not enough itself and individuals need external support to transform efficacy into actions. Although the empirical literature on the family support role is limited, its value has been highlighted in the past (e.g., Altinay et al., 2012; Hsu et al., 2019). While identifying the role of family support, Searman et al. (2016) commented that family role is always needed to an entrepreneur and all phases of the process should be backed by external factors like family support. Klyver (2007), on the other hand, valued the role of family support in the idea generation phase, as emotional support is the most important form of support offered by the family.

While looking at the buffering effects of family support in ESE and P-E fit, the limited literature called us to work on this area. While looking at the P-E fit, it’s considered as the perception of fit is only possible when one believes that he/she can meet the job requirements (high self-efficacy could predict that), but external support may buffer this belief (i.e., verbal persuasion, Bandura and Walters, 1977). As the family is a source of encouragement (Lotfizadeh and Heidarzadeh Hanzaee, 2014; Stamboulis and Barlas, 2014; Edelman et al., 2016) and external factor are equally valued influencing one’s belief and intentions (Saks and Ashforth, 1997; Kristof‐Brown et al., 2005), it’s assumed that both in presence of family support the relationship of self-efficacy with outcomes will be strengthened. van Vianen (2018) work on Person-environment fit also highlighted that both environment and person predict human behavior. Based on the premise of van Vianen (2018) we also assumed that person-entrepreneurship fit will not only be influenced by personal factors but environmental factors like family support may foster the outcomes. These findings highlight an important phenomenon prevalent in poor countries like Pakistan.

### Implications of the study

This study adds value in the existing body of knowledge by exploring the mechanism of ESE and intentions relation through largely ignored roles of family support and P-E fit. The findings of the study extend the body of knowledge on the person-environment fit model of entrepreneurship (Markman and Baron, 2003). Additionally, this study offers the explanatory mechanism of internal and external factors (Hsu et al., 2019), in tandem, that influences the entrepreneurial intentions and behaviors. As the sample of the study consisted of students that are going to make their career choices in the future, the study of intentions was well in time. Future researchers extend this study by considering other internal and external factors in the model. For instance, locus of control could be an important consideration as an individual with an internal locus of control may not take the environmental/external factors as supportive and desirable (Li et al., 2015). Thus the external forces (e.g., family support) may not have buffering effects. Future studies could also consider the role of other forms of support (e.g., institutional support, educational institution support), credit facility (Qunlian, 2011). Personality could also be an important determinant of fit and intentions, as big five personality traits influence intentions (e.g., Qunlian, 2011), but its link with P-E fit is largely left unattended. Dark personality triad could also be investigated, as the narcissist individuals may not see others favorably as they remain in the state of self-liking and admiration (Tucker et al., 2016). Future studies should also consider the entrepreneurial behaviors instead of intentions, as individuals with high ESE and P-E fit may have more behavioral outcomes.

This study does not only carry theoretical importance but it is equally good at its empirical implications. The findings highlight the role of internal and environmental variables in predicting perceptions and intentions for entrepreneurship. Thus may help institutions, parents, and individuals who may have adopted an entrepreneurial career. It will also be useful for those who have to mentor, guide or coach someone for an entrepreneurial career. Thus, conclusively, the study explains the mechanism of personality attributes and entrepreneurial intentions and offers a novel explanation with future directions to work.

### Limitations and future directions

Though the study is carried out using a sound methodology and rigorous analytical technique, it is still prone to some limitation. To start with the cross sectional design with sample from students, influences the depth as longitudinal studies and sample of intrapreneurs may portray a better picture of entrepreneurship in a society. The current study can be extended further by future researchers, for instance, one consideration be to study the determinants of ESE in the model which may include entrepreneurial education, ability to identify opportunities (Hassan et al., 2020; Anwar et al., 2022), perceived university support (Liu et al., 2022) and entrepreneurial passion (Neneh, 2022). Future studies should also consider the boundary condition of entrepreneurial education which has strongly bearing on one’s career choices, intentions and actions (Anwar et al., 2020). Another important consideration could be on the mechanism between intentions and its predictors, e.g., passion, motivation, and attitude (Anwar et al., 2021). Future studies should also consider the social and job support at P-Intrapreneurship fit perspective of employees working in organization. According to Bogatyreva et al. (2022), observed that “Conducive institutions stimulate individual involvement in intrapreneurship” (p. 45). It is therefore believed that P-Int fit is an area requiring researchers’ attention.

## Data availability statement

The datasets presented in this article are not readily available due to issues of confidentiality and anonymity. Requests to access the datasets should be directed to ishfaqahmed@hcc.edu.pk.

## Author contributions

The author confirms being the sole contributor of this work and has approved it for publication.

## Conflict of interest

The author declares that the research was conducted in the absence of any commercial or financial relationships that could be construed as a potential conflict of interest.

## Publisher’s note

All claims expressed in this article are solely those of the authors and do not necessarily represent those of their affiliated organizations, or those of the publisher, the editors and the reviewers. Any product that may be evaluated in this article, or claim that may be made by its manufacturer, is not guaranteed or endorsed by the publisher.
